# Multi-Perspective Hierarchical Deep-Fusion Learning Framework for Lung Nodule Classification

**DOI:** 10.3390/s22228949

**Published:** 2022-11-18

**Authors:** Kazim Sekeroglu, Ömer Muhammet Soysal

**Affiliations:** 1 Department of Computer Science, Southeastern Louisiana University, Hammond, LA 70402, USA; 2 School of Electrical Engineering and Computer Science, Louisiana State University, Baton Rouge, LA 70803, USA

**Keywords:** computer-aided detection, lung cancer, deep learning, hierarchical learning, hierarchical fusion, convolutional neural networks, modular training and modular learning

## Abstract

Lung cancer is the leading cancer type that causes mortality in both men and women. Computer-aided detection (CAD) and diagnosis systems can play a very important role for helping physicians with cancer treatments. This study proposes a hierarchical deep-fusion learning scheme in a CAD framework for the detection of nodules from computed tomography (CT) scans. In the proposed hierarchical approach, a decision is made at each level individually employing the decisions from the previous level. Further, individual decisions are computed for several perspectives of a volume of interest. This study explores three different approaches to obtain decisions in a hierarchical fashion. The first model utilizes raw images. The second model uses a single type of feature image having salient content. The last model employs multi-type feature images. All models learn the parameters by means of supervised learning. The proposed CAD frameworks are tested using lung CT scans from the LIDC/IDRI database. The experimental results showed that the proposed multi-perspective hierarchical fusion approach significantly improves the performance of the classification. The proposed hierarchical deep-fusion learning model achieved a sensitivity of 95% with only 0.4 fp/scan.

## 1. Introduction

Although lung cancer is the second most commonly diagnosed cancer in both men and women, it is the leading cancer type that causes mortality in both men and women [1]. Lung nodule detection is a very challenging task. The research team in [2] explored the effect of low-dose CT scans on cancer mortality. Utilizing either low-dose CT or chest radiography, they screened around 53 K high lung cancer risk patients three times a year between August 2002 and April 2004. The results of their study show that there is a 20% reduction in mortality of the patients who were screened by low-dose CT scan. Even though CT scans help to reduce the mortality rate, the radiologists’ decisions may differ significantly in the identification of lung nodules from the CT scans. As an example, ref. [3] shared the results of two radiologists’ examination over 25 CT scans; the results show that one of the radiologists detected 20 nodules, whereas the other radiologist detected 63 nodules from the same CT scans.

A CAD system increases the performance of nodule detection substantially. The study conducted by [4] showed that the CAD system significantly reduced the number of false positives (FPs). The research by [5] studied the effect of a CAD system in the detection of small nodules shared the results of six radiologists’ examinations over 52 CT scans with/without a CAD system. The results show that the CAD system improves a radiologist’s performance considerably. In [6], the performance of the commercial CAD software Lung-CAD VB10 A and Siemens AG Healthcare was compared with the performance of two independent readers for detecting the pulmonary nodules in the NELSON dataset. The study showed that the sensitivity of CAD was 96.7% with a 3.7 FPs/scan and the sensitivity of the double reader was 78.3% with 0.5 FPs/scan. Therefore, CAD systems with a higher nodule detection rate can be a good help for radiologists to decrease the number of missed nodules, particularly, the small nodules in their early stages.

**Figure 1:**
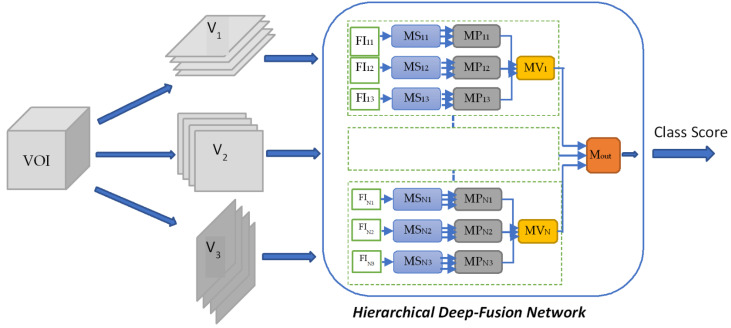
The proposed hierarchical deep-fusion framework.

In this study, we propose a hierarchical deep-fusion learning method utilizing multiple views of 3D spatial data. The proposed framework is seen in Figure 1. Once the volume of interest (VOI) is extracted, the slices from different perspectives are fed into the hierarchical deep fusion network, and the class scores are computed and fused in a hierarchical manner. Different types of fusion schemes are proposed in hierarchical deep-fusion networks. The proposed basic scheme is the multi-perspective hierarchical fusion of raw images (MPF) where the slices from different perspectives are classified hierarchically and the class scores are fused at the decision level by the proposed supervised learning-based fusion method. Another proposed fusion schema is based on a single feature image, and it is called single feature multi-perspective fusion (SFMPF) in which the feature images are used as an input to the hierarchical deep fusion network. The single-feature image approach is used to extend the basic MPF scheme to a multi-feature and multi-perspective fusion (MFMPF) by using different types of feature images from different perspectives and fusing them with the proposed hierarchical fusion approach. MFMPF scheme allows to fuse decisions made by looking at different features and different perspectives of the 3D object.

Contributions of this study can be summarized as (a) utilizing deep learning in a multi-view hierarchical decision-making scheme, (b) proposing a supervised learning-based fusion method to be used in this hierarchical scheme, (c) introducing a modular training approach for the hierarchical scheme, (d) utilizing feature images in the proposed hierarchical deep-fusion learning, and (e) adding another level of hierarchy to the proposed model by fusing the multiple feature image-based hierarchical deep-fusion learning models. Whereas there are limitations of this study such as not being invariant to 3D rotation, having a limited size, and the variation of the dataset to explore the proposed model. Since the proposed architecture is based on CNNs, they are not invariant with rotation. Therefore, the proposed method is not invariant with 3D rotation. In addition, the size of the dataset can be increased as well as the proposed model can be explored by training and testing with a different dataset.

The rest of the paper is organized as follows: In the following section, the previous work on lung nodule detection is provided. The third chapter introduces the proposed hierarchical deep-fusion learning models. In chapter four, data preparation, experimental results, and discussions are provided. Finally, conclusions with the feature directions of the proposed research are covered in chapter five.

## 2. Related Works

Computer-aided detection and diagnosis (CAD) systems have been studied for decades to get more accurate detection and to decrease the workload on radiologists. Complete computer-aided detection and diagnosis algorithms are usually composed of three main blocks: (1) Detection of the nodule candidates, (2) extraction of the features from the nodule candidates, and (3) false-positive reduction and classification. Different approaches are used for the detection of the nodule candidates based on 2D or 3D segmentation. Since the intensity value of the nodule and the other structures in the lung region differ from each other, most of the segmentation methods are based on gray-level thresholding. After segmenting out the nodule candidates, the next step is extracting the robust features for classification. The most common features extracted from the nodules are shape and texture-based features. Once the features are extracted from the nodule candidates, to reduce the false positives, one of the classification methods such as k-nearest neighbor, support vector machine, linear discriminant, or random forest classifier is used.

In [7] after detecting the nodule candidates, local image features; number of voxels, compactness, ratio, and sphericity are used with 2 stage k-NN classifier for the false positive reduction. Eight hundred and thirteen CT scans from NELSON Trial data generated in Europe were used and the proposed method achieved a sensitivity of 80% with an average of 4.2 false positives per scan (FPs/scan). In [8] a fully automated CAD system for lung nodule detection algorithm is proposed. The authors state that detecting and segmenting the nodules at the same time is one of the advantages of their candidate detection algorithm. Once the nodule candidates are detected, a total of 245 features based on geometric, intensity, and gradient are extracted from each nodule candidate. A sequential forward selection process is used to select the best descriptive features from out of 245 features and these features are used in Fisher linear discriminant (FLD) classifier and a quadratic classifier. The comparison result of the two classifiers shows that the FLD classifier performs better than the quadratic classifier. According to the 7-fold cross-validation, the sensitivity of the proposed CAD system with the FLD classifier is 82.66% with an average of 3 FPs/scan using the LIDC dataset. There are 84 scans and 143 nodules in the LIDC data set. 

In [9], the authors aim to develop a CAD system that can automatically detect a pulmonary nodule greater than or equal to 3 mm. Once they segmented the nodule candidates by using 3D mass-spring models, seven features: 1. Surface area, 2. Volume, 3. Sphericity, 4. Mean of the nodule intensity, 5. Standard deviation of the nodule intensity, 6. Skewness of the nodule intensity, and 7. Kurtosis of the nodule intensity are extracted from each nodule candidate. They have two stages for false positive reduction and classification. In the first stage, they are eliminating the noodles candidates smaller than 3 mm and greater than 50 mm. In the second stage, they are using a neural network with one input, one hidden, and one output layer for the classification of nodule candidates. They have 84 CT scans from the LIDC dataset and 148 nodules. The proposed algorithm reaches a sensitivity of 88% with 2.5 FPs/scan. In [10] as in most CAD systems, the proposed method also has two main stages, nodule candidate detection, and false positive reduction. A hierarchical 3D block analysis method is used for nodule detection and an SVM classifier is used for false-positive reduction. After nodule candidates are detected, 2D and 3D geometric features such as area, diameter, circularity, volume, compactness, elongation, and 2D texture features such as the mean, variance, skewness, kurtosis, and eigenvalues are extracted to be used as an input to the SVM classifier. The LIDC dataset is used for the experiments. There are 84 scans in the LIDC dataset but only 58 of them contains nodule and only those 58 scans were used in the experiments. The proposed method achieved 95.28% sensitivity with 2.27 FPs/scan.

Ref. [11] concentrate on computer-aided detection of subsolid pulmonary nodules. The authors used a threshold-based method for nodule candidate detection. Once the nodule candidates are detected, a set of 128 features based on intensity, texture, shape, and context are extracted from each of the nodule candidates. Then, these features are used in different types of classifiers such as GentleBoost, SVM, k-nearest neighbor, linear discriminant, nearest mean, and random forest classifiers. According to the results from the FROC curves, the GentleBoost classifier performs best, and it reaches a sensitivity of 80% with 1 FPs/scan.

In most of the proposed CAD algorithms, the data set used in training and testing and the way the performance is assessed differ from method to method. Therefore, there is a bottleneck in the comparison between the performances of the proposed CAD algorithms [12]. There are few studies which compares the performance of the existing CAD systems by using the same dataset and the same evaluation method. 

In [13], existing CAD methods were compared by testing and evaluating them with the same data and the same method, and also the authors proposed a method for combining the tested CAD system for a better performance. In this study ANODE09 database which includes 55 scans from a lung cancer screening program is introduced. The performance of the six different CAD algorithms was compared and each CAD method was evaluated based on their average sensitivity of seven different FP rates: 1/8, 1/4, 1/2, 1, 2, 4, and 8. According to the results, there is a significant performance difference between the algorithms and combining the results of each CAD system leads to a better performance.

Another study to improve the performance of the existing CAD system by combination is proposed by [14]. They propose a set of four different methods to combine the existing CAD systems for four different scenarios for a better performance. The first method is proposed where there is only the location of the nodule is available as an output of the CAD system. In this case, the method suggests combining the detected locations of the CAD systems. In the second and third scenarios, in addition to the location of the nodule, the level of suspicion for each detected nodule is available. Lastly in the fourth scenario, most of the internal details such as training data, feature vectors, classifiers, etc., of the CAD systems are available. However, the authors did not discuss a combination method for this case. Since it is not likely to have access to the internal details of most of the CAD systems in practice. 

In [15], the performance of the state-of-the-art CAD systems VISIA, Herakles, and ISICAD for detection of the pulmonary nodules is compared by using the LIDC/IDRI dataset. After a comparison of the CAD systems, the false positives of the best-performing one were examined by four radiologists to see if the CAD system can detect any nodule that was missed by the radiologist during the annotation. Out of these three CAD systems, Herakles performed best with a sensitivity of 82% with 3.1 FPs/scan for nodules annotated by all four readers. While Herakles achieved a more robust performance, the other two CAD systems VISIA and ISICAD showed substantial performance differences on the LIDC/IDRI dataset. The reason for the performance drop on ISICAD is that it is trained exclusively on the NELSON dataset which “consists of homogeneous thin-slice data reconstructed with a soft/standard reconstruction kernel”. Thus, it is important to use heterogeneous datasets such as LIDC/IDRI to train and test the CAD system. Lastly, there were 45 nodules which were accepted as nodule ≥ 3 mm by all four radiologists detected by the CAD system but overlooked by the radiologist during the annotation procedure.

According to the review of CAD systems for lung cancer in CT scans, CAD systems are still not used widely by the community of radiologists. Therefore, further research and development is needed in CAD systems, particularly for decreasing the “number of false positives (FP), having high processing speed, presenting high level of automation, low cost (of implementation, training, support, and maintenance), the ability to detect different types and shapes of nodules, and software security assurance” [16].

The state-of-the-art computer vision methods for object detection are based on deep learning methods. Therefore, there are existing CAD algorithms for pulmonary nodule detection which are based on deep learning methods such as convolutional neural networks, deep belief networks, and autoencoders. One of the earliest studies that uses a deep learning system for lung nodule classification is [17]. In [17], the classification of the pulmonary nodules as being malignant or benign by using deep learning methods was explored. Specifically, the deep belief network (DBN) and convolutional neural network (CNN) models were tested. This is one of the first studies that explores the application of deep learning techniques for the classification of pulmonary nodules. The LIDC-IDRI dataset includes 1010 scans and 2545 nodules which are greater than 3 mm are used for testing the proposed methods in [17]. For the comparison of deep learning methods and the feature-based methods, two of the well-performing features SIFT and local binary pattern (LBP) features with k-NN classifier are used. DBN was able to classify pulmonary nodules with 82.2% sensitivity and the SIFT+LBP feature-based classifier reached a sensitivity of 66.8%. Another earlier study for classifying pulmonary nodules as malignant or benign is [18]. The classification is performed by using the deep features extracted from 2D images by the autoencoder and classified by the binary decision tree. The publicly available LIDC/IDRI dataset is used to train and test the algorithm. Although there are 1010 CT scans available in LIDC/IDRI dataset, only 157 scans have the proper annotation for the nodules being benign or malignant. The proposed method achieved a sensitivity of 83.35% with 0.39 FPs/scan over a 10-fold cross-validation. 

In [19], a 3D convolutional neural network-based lung nodule classification algorithm is proposed. The authors state that the proposed method can work with weakly labeled 3D data as in the case of only the label of the central voxel and the size of the largest expected nodule are provided. Once they estimate the labels of the 3D training data by using basic thresholding and simple linear iterative clustering (SLIC) [20] super-pixels of the 2D slices, they use this data to train 3D CNN for nodule classification. The negative samples are extracted from the lung area by randomly sampling the locations based on the threshold. The SPIE-AAPM-LUNGx dataset is used to train and test the proposed method. The dataset contains 70 CT scans. Moreover, 15 K positive and 20 K negative samples are labeled by the proposed method. The proposed method achieved 80% sensitivity with 10 FPs/scan.

Another study that uses deep learning methods for lung nodule detection is [21]. The authors proposed a multi-view CNN for lung nodule detection. In the proposed method, they are extracting the volume of interest as a cube. Then, 2D patches from nine symmetrical perspectives of the extracted volume are fed into separate CNNs. The outputs of the CNNs are fused in three different approaches. The first fusion approach is called the committee fusion where the fusion is conducted at the decision level. Once the class scores from each CNN are computed, class scores are fused using a product rule on the output probabilities [22]. The second fusion approach is late fusion where the fusion is performed at the feature level by concatenating the outputs of the first fully connected layers. Lastly, in the third fusion approach, they are using mixed fusion which is the combination of the committee and late fusion. Although this proposed method is fusing the slices from multi-view, they are using a single slice from each view, and the way the fusion is performed is similar to the previously proposed fusion approaches whereas our study proposes a hierarchical deep-fusion based on modular training and supervised learning. 

The researchers in [21,23,24] reported that fusion-based models increase the performance of classification. In general, averaging, multiplication, or voting schemes are employed as a fusion method in deep learning [24,25,26,27]. These strategies utilize a simple approach to reach a final decision out of multiple predictions. Among few studies that explore deep learning for lung nodule classification, the only method that uses multiple perspectives of a volume is proposed in [21]. However, the method utilizes only a single slice from each perspective and has only one level of simple fusion. In contrast, this research proposes a new kind of ensemble learning strategy “hierarchical deep-fusion learning” that aims to learn gradually from in-parallel and prior predictions obtained from different views.

## 3. Method

### 3.1. Multi-Perspective Hierarchical Deep-Fusion Learning Model (MPF)

**Figure 2:**
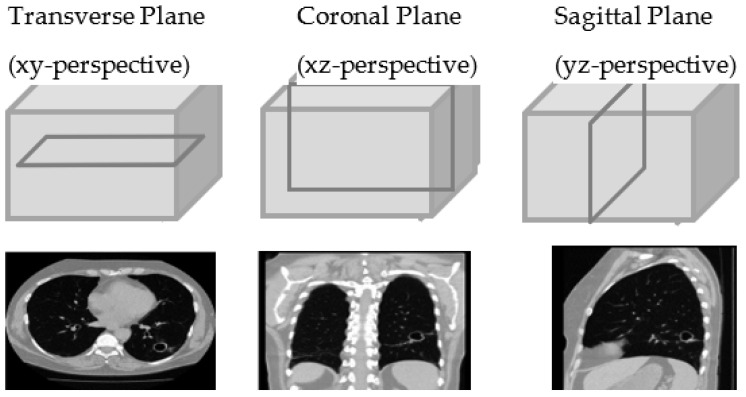
Slices from three different perspectives.

In this paper, we propose a hierarchical deep-fusion learning scheme [28]. In the proposed method, there are three levels of hierarchical predictions: (1) Slice level, (2) perspective level, and (3) volume level. We employed three different perspectives: transverse, coronal, and sagittal as shown in Figure 2.

**Figure 3:**
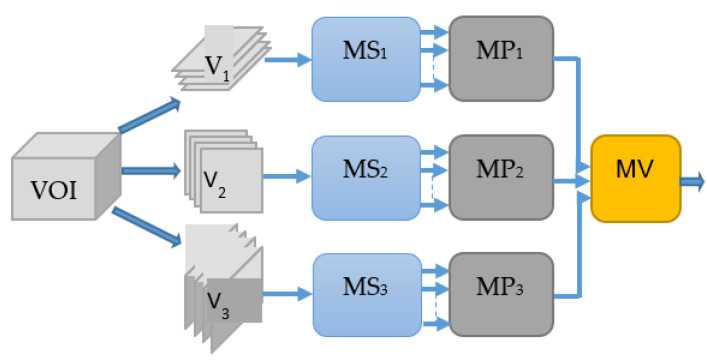
Block diagram of the proposed model.

**Figure 4:**
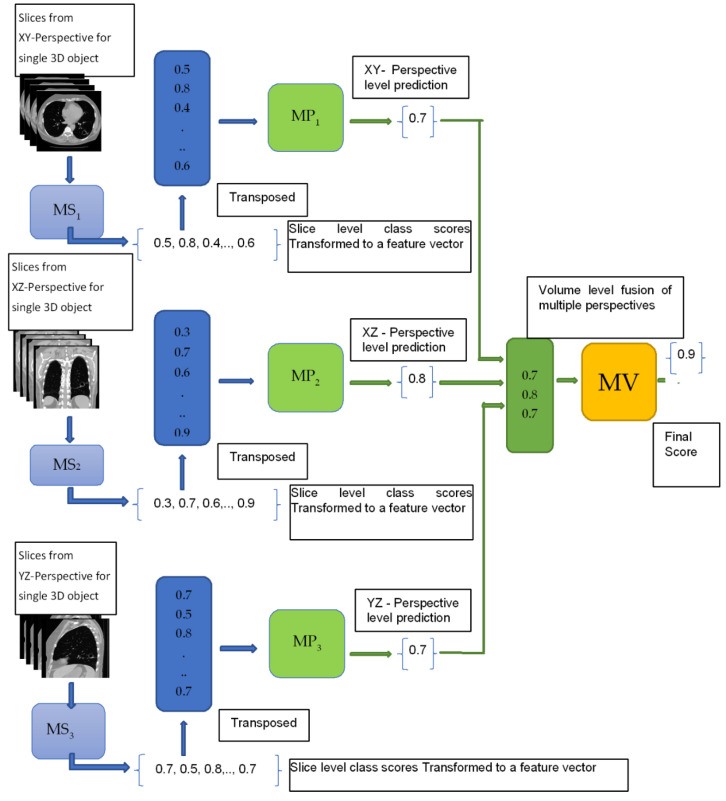
Re-arraignment of the class scores from slice level classification to create an input feature for the perspective module.

The block diagram of the proposed hierarchical deep-fusion learning scheme is illustrated in Figure 3. In the proposed method, each module at each level is trained separately in a hierarchical modular fashion; that is, the decision made at each level is predicted based on the decision from the previous layer. The proposed hierarchical learning process is as follows: Once a volume of interest is extracted from an object, a stack of 2D slices for each perspective is generated; the same VOI is represented by three sets of 2D slices, namely V_1_, V_2_, and V_3_. At the first hierarchical level, a decision is produced for each slice of its perspective by a slice module (MS). Therefore, there are three slice modules MS*_i_* at the first hierarchical level, one per perspective V*_i_*. At the following second level, another learning module, namely the perspective module (MP), is employed. An MP*_i_* reformats the class scores obtained for each slice of V*_i_* to form its input feature vector. As an example, assuming each stack V*_i_* has 10 slices, the MS*_i_* produces 10 class scores. These scores are formed into an input feature vector of size 10 by 1 for MP*_i_*. At the second level, each MP*_i_* predicts a single class score for each V*_i_*. Similarly, the output scores of MPs are reformatted to form an input feature vector of size 3 by 1 for the last level module, namely the volume module (MV), of the hierarchical scheme. The MV computes the final decision for the volume of interest (VOI). The block diagram of the re-arrangement process of the class scores is provided in Figure 4.

**Figure 5:**
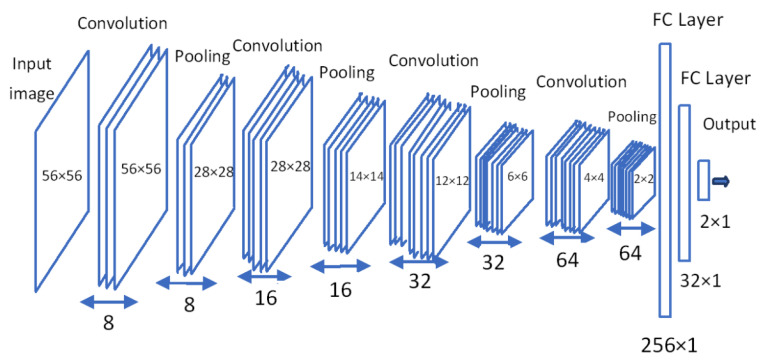
DCNN structure.

In the proposed hierarchical deep learning scheme, a slice module MS*_i_* is a deep convolutional neural network (DCNN) as illustrated in Figure 5. Although the structure of each MS*_i_* is the same, they are trained separately. The DCNN structure consists of four convolutional and four pooling layers followed by the regular one-hidden layer feed-forward neural network. The input size of each 2D slice is 56 × 56 pixels. At the first convolution layer, there are 8 filters in the size of 3 × 3. The number of filters at the second, third, and fourth convolutional layers are double the number of filters at their previous convolutional layers. Hence, there are 64 filters at the last convolutional layer. After the last pooling layer, there is a fully connected layer comprised of 32 neurons. At the convolutional and fully connected layers rectified linear function defined by (1) and at the output layer SoftMax function defined by (2) are used. The filters at each convolution layer are adjusted by back-propagating the error obtained at the output based on the cross-entropy loss function defined by (3).

(1) $$f\left(x\right)=\max\left(0,x\right)$$

(2) $$\sigma {\left(x\right)}_{j}=\frac{e^{x_{j}}}{\sum_{k=1}^{K}e^{x_{k}}}$$

where *K* is the total number of neurons in the layer, and *j* is the index of the neuron at the output layer.

(3) $$L=-\underset{j}{\sum }t_{j}\log\left(p_{j}\right)$$

where *t* is the target, *p* is the predicted values at the output layer, and *j* is the index of the neuron at the output layer.

In the perspective module and the volume module, a supervised classifier such as support vector machine, ANN, Bayesian network, or a multi-dimensional regression model can be used. In this study, a regular feedforward ANN is used for the perspective and the volume level predictions.

### 3.2. Single Feature & Multi-Perspective Hierarchical Deep-Fusion (SFMPF)

**Figure 6:**
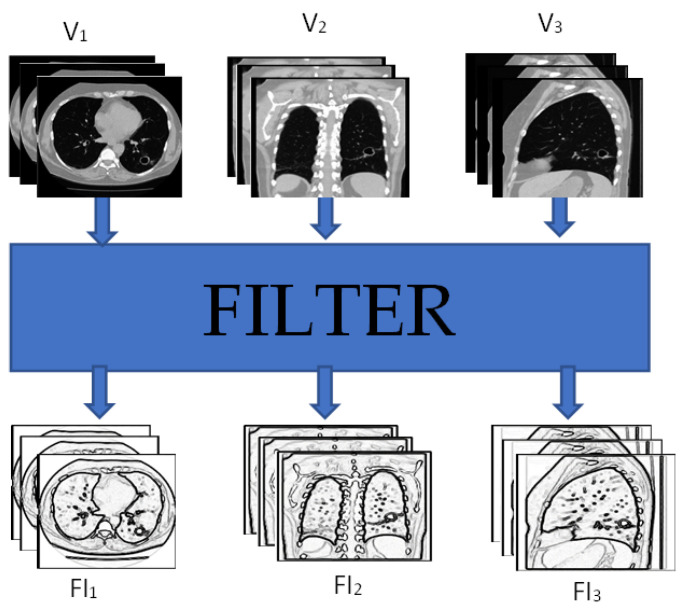
Feature images are created by filtering the slices from each perspective.

**Figure 7:**
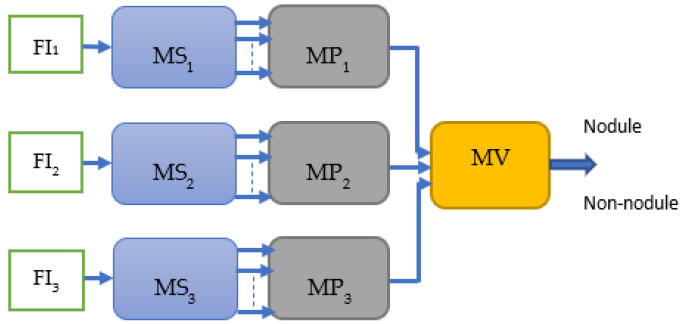
Block diagram of the proposed SFMPF model.

Extracting salient content from the input data can lead to a better representation and better classification accuracy. Therefore, in the proposed method, feature images are used instead of raw images to learn the representation of the data. Features images can be obtained by applying filters such as Bilateral, Trilateral, LOG, or Gabor filters to the raw images as shown in Figure 6 where V_1_, V_2_, and V_3_ are the raw images from three different perspectives and FI_1_, FI_2_, and FI_3_ are the feature images. Once the feature images are created, they are fed into the same proposed hierarchical fusion network architecture as shown in Figure 7.

#### Creating Feature Images

In the proposed feature-based hierarchical deep fusion, 4 different methods, Bilateral filtering, Trilateral filtering, Laplacian of Gaussian (LoG) filtering, and Gabor filtering, are used to produce the feature images. In this section, backgrounds for LoG, Gabor, Bilateral, and Trilateral filters, are provided.

Laplacian operator (Δ) can be used to measure the rapid changes in the image. Laplacian of an input image $I\left(x,y\right)$ at a pixel point $\left(x,y\right)$ is given by (4)

(4) $$\Delta I\left(x,y\right)=\frac{\partial^{2}I}{\partial x^{2}}+\frac{\partial^{2}I}{\partial y^{2}}$$

However, before applying the Laplacian operator, smoothing the input image to reduce the effect of noise is a very common approach. Therefore, the input image is convolved with a Gaussian filter, defined by (5), with the shape parameter σ before applying the Laplacian operator as in (6).

(5) $$G\left(x,y,\sigma \right)=\frac{1}{2\pi \sigma^{2}}e^{-\left(x^{2}+y^{2}\right)/2\sigma^{2}}$$

(6) $$\Delta \left[G\left(x,y,\sigma \right)*I\left(x,y\right)\right]$$

To reduce the cost of computation, one can use (7) instead of (6)

(7) $$\Delta \left[G\left(x,y,\sigma \right)*I\left(x,y\right)\right]=\Delta G\left(x,y,\sigma \right)*I\left(x,y\right)$$

where (7) is derived using the convolution property defined by (8).

(8) $$\frac{d}{dt}[h\left(t\right)*f\left(t\right)=\frac{d}{dt}\int f\left(\tau \right)h\left(t-\tau \right)d\tau =\int f\left(\tau \right)\frac{d}{dt}h\left(t-\tau \right)d\tau =f\left(t\right)*\frac{d}{dt}h\left(t\right)$$

Hence the LoG $\Delta G\left(x,y,\sigma \right)$ is given by

(9) $$\Delta G\left(x,y,\sigma \right)=-\frac{1}{\pi \sigma^{4}}\left[1-\frac{x^{2}+y^{2}}{2\sigma^{2}}\right]e^{-\frac{x^{2}+y^{2}}{2\sigma^{2}}}\;$$

**Figure 8:**
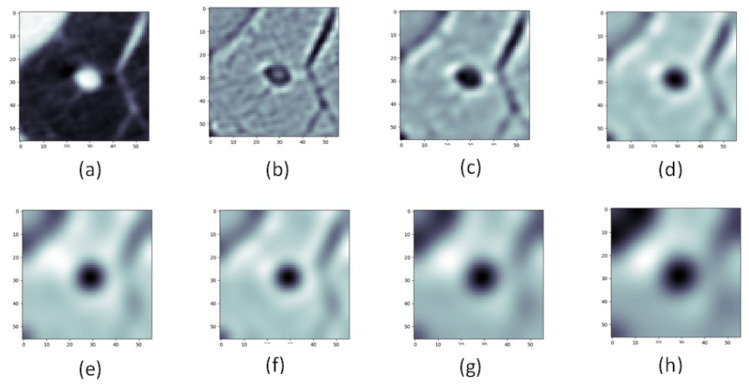
(**a**) Raw nodule, (**b**) through (**h**) are LoG filtered nodule with σ=1 to σ=7 by 1 incremental.

Figure 8 shows the feature image obtained by filtering the nodule employing the LoG filter using different σ values.

In addition to the LoG filter, the Gabor filter is also used to create the feature image. Gabor filters are typically used to extract the textures in the images. The Gabor filter is composed by multiplying a Gaussian kernel with a complex sinusoid.

(10) $$G\left(x,y\right)=g\left(x,y\right)\;s\left(x,y\right)$$

where $g\left(x,y\right)$ is a 2D Gaussian kernel with the standard deviation of $\sigma_{x}$ and $\sigma_{y}$,

(11) $$g\left(x,y\right)=\frac{1}{2\pi \sigma_{x}\sigma_{y}}e^{-\frac{1}{2}\left(\frac{x^{2}}{\sigma_{x}^{2}}+\frac{y^{2}}{\sigma_{y}^{2}}\right)}$$

and $s\left(x,y\right)$ is the complex sinusoid with the center frequency of $\omega_{x_{0}}$ and $\omega_{y_{0}}$

(12) $$s\left(x,y\right)=\cos\left(2\pi \omega_{x_{0}}x+2\pi \omega_{y_{0}}y\right)+isin\left(2\pi \omega_{x_{0}}x+2\pi \omega_{y_{0}}y\right)$$

Using the Euler’s formula (13)

(13) $$e^{i\theta }=\cos\theta +isin\theta$$

(12) can be written as

(14) $$s\left(x,y\right)=e^{i\left(2\pi \left(\omega_{x_{0}}x+\omega_{y_{0}}y\right)\right)}$$

Therefore, the complex Gabor filter is

(15) $$G\left(x,y\right)=\frac{1}{2\pi \sigma_{x}\sigma_{y}}e^{-\frac{1}{2}\left(\frac{x^{2}}{\sigma_{x}^{2}}+\frac{y^{2}}{\sigma_{y}^{2}}\right)}e^{i\left(2\pi \left(\omega_{x_{0}}x+\omega_{y_{0}}y\right)\right)}$$

The real part of the Gabor filter is

(16) $$G\left(x,y\right)=\frac{1}{2\pi \sigma_{x}\sigma_{y}}e^{-\frac{1}{2}\left(\frac{x^{2}}{\sigma_{x}^{2}}+\frac{y^{2}}{\sigma_{y}^{2}}\right)}\cos\left(2\pi \omega_{x_{0}}x+2\pi \omega_{y_{0}}y\right)$$

The imaginary part of the Gabor filter is

(17) $$G\left(x,y\right)=\frac{1}{2\pi \sigma_{x}\sigma_{y}}e^{-\frac{1}{2}\left(\frac{x^{2}}{\sigma_{x}^{2}}+\frac{y^{2}}{\sigma_{y}^{2}}\right)}\sin\left(2\pi \omega_{x_{0}}x+2\pi \omega_{y_{0}}y\right)$$

A Bilateral filtered image at a pixel c within an N-by-N neighbor is defined as

(18) $$I_{B}\left(c\right)=\frac{1}{W}\overset{c+N/2}{\underset{r=c-N/2}{\sum }}B\left(r;c,\sigma_{S},\sigma_{R}\right)\;I\left(r\right)$$

where $\sigma_{S}$ and $\sigma_{R}$ are the shape parameters of the spatial Gaussian filter S and the range (intensity) Gaussian filter R, respectively, and the Bilateral filter that operates at the central pixel c and the neighboring pixels r is defined by

(19) $$B\left(r;c,\sigma_{S},\sigma_{R}\right)=S\left(r;c,{\;\sigma }_{S}\right)\;R\left(I\left(r\right);I\left(c\right),\sigma_{R}\right)$$

(20) $$S\left(r;c,{\;\sigma }_{S}\right)=e^{\frac{-{\left|\left|r-c\right|\right|}^{2}}{2\sigma_{S}^{2}}}\;R\left(I\left(r\right);I\left(c\right),\sigma_{R}\right)=e^{\frac{-{\left[I\left(r\right)-I\left(c\right)\right]}^{2}}{2\sigma_{R}^{2}}}$$

(21) $$W=\overset{c+N/2}{\underset{r=c-N/2}{\sum }}S\left(r;c,{\;\sigma }_{S}\right)\;R\left(I\left(r\right);I\left(c\right),\sigma_{R}\right)$$

The normalization factor W ensures the sum of the weights is one. The range filter $R\left(I\left(r\right);I\left(c\right),\sigma_{R}\right)$ determines the effect of the neighbor pixels on the central pixel value $I\left(c\right)$ during the smoothing operation. In other words, the contribution of the neighbor pixels is adaptively determined. This adaptive characteristic of the Bilateral filter preserves high-frequency structures such as edges during the smoothing operation. Note that the spatial filter $S\left(r;c,{\;\sigma }_{S}\right)$ penalizes a neighbor pixel more when it moves away from the center while the range filter penalizes a pixel more when its intensity differs more from the central pixel intensity.

We proposed a new filter called the Trilateral filter [29]. This filter is formed by adding a Laplacian kernel as a second range kernel in addition to spatial and range kernels in the Bilateral filter. This additional anisotropic filter measures the variation of the gradient and adjusts the weight of neighbor pixels accordingly. The Trilateral filter is more effective at higher frequency structures. Hence, the high-frequency content is preserved. On the other hand, the range kernel of the Bilateral filter only compares the intensity difference; therefore, it does not penalize as much as the Trilateral filter. Over a lower frequency region, the Bilateral and Trilateral filters behave similarly. As an alternative to a Laplacian kernel, a gradient kernel would be used; however, the Laplacian kernel has the advantage to measure the speed of the gradient, which is important in high-frequency regions. Similar to the Bilateral filter, the Trilateral filtered image is defined by

(22) $$I_{T}\left(c\right)=\frac{1}{W}\overset{c+N/2}{\underset{r=c-N/2}{\sum }}T\left(r;c,\sigma_{S},\sigma_{R},\sigma_{L}\right)\;I\left(r\right)$$

(23) $$T\left(r;c,\sigma_{S},\sigma_{R},\sigma_{L}\right)=\;S\left(r;c,\sigma_{S}\right)\;R\left(\Delta I\left(r;c\right);\sigma_{R}\right)\;L\left(\Delta \nabla^{2}I\left(r;c\right);\sigma_{L}\right)$$

where $\sigma_{L}$ is the shape parameter of the Laplacian kernel L(*). The difference of Trilateral images $D_{T}$ to construct the scale-space for the extrema detection is given by the following equation:

(24) $$D_{T}\left(x,y,\sigma_{S},\sigma_{R},\sigma_{L}\right)=\left[T\left(x,y,{k\;\sigma }_{S},\sigma_{R},\sigma_{L}\right)-T\left(x,y,\sigma_{S},\sigma_{R},\sigma_{L}\right)\right]*I\left(x,y\right)$$

### 3.3. Multi-Feature & Multi-Perspective Hierarchical Deep-Fusion (MFMPF)

**Figure 9:**
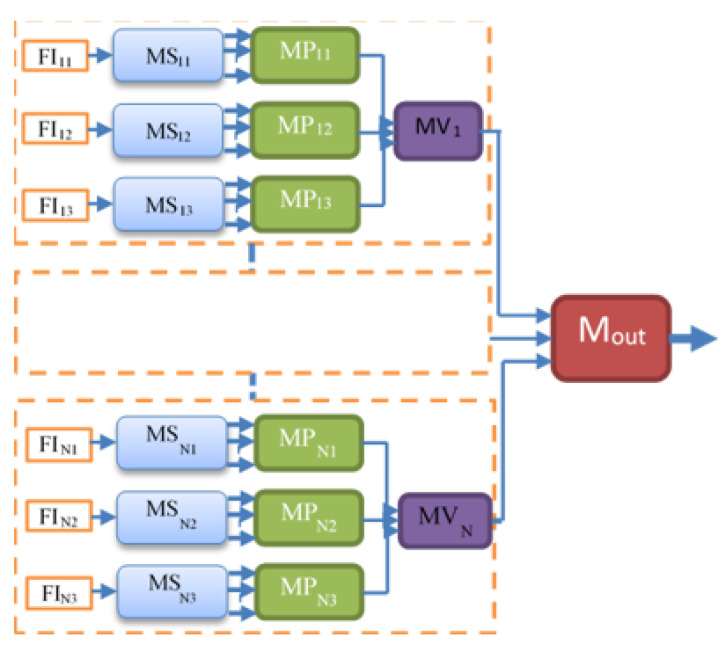
Block diagram of MFMPF model.

Figure 9 the block diagram of the hierarchical fusion of multiple SFMPF models is illustrated. This proposed fusion scheme is the composition of the previously proposed SFMPF scheme with different types of feature images. The idea is first to make multiple decisions by different SFMPF models which make the predictions based on different types of feature images. Then, the decisions obtained from the multiple SFMPF models are fused to make the final decision by utilizing an additional hierarchical layer. In this study, the proposed MFMPF model predicts a class score by fusing the decisions from four different SFMPF models based on Bilateral, Trilateral, Laplacian of Gaussian (LoG), and Gabor filtering.

## 4. Experiments and Results

### 4.1. Data Preparation

#### 4.1.1. Data

Publicly available lung CT scan database created by The Lung Image Database Consortium (LIDC) and Image Database Resource Initiative (IDRI) [30] is used to test the proposed CAD framework. The LIDC/IDRI database contains 1010 CT scans which have the annotations for the nodules and the non-nodules which has diameter ≥ 3 mm. Annotations made by the radiologists belong to one of these three groups; nodule ≥ 3 mm, nodule ≤ 3 mm, or non-nodule ≥ 3 mm. CT scans are annotated by 4 expert radiologists in 2 phases, the blinded-read phase and the unblinded-read phase. In the initial blinded-read phase, each of the radiologists examined the scans independently without knowing the opinion of the others, and in the second unblinded-read phase, they examined the CT scans while knowing the annotations made by 3 other radiologists. While the surrounding boundary for the nodules ≥ 3 mm is annotated, the nodules ≤ 3 mm or non-nodules ≥ 3 mm have only their volume center annotated. In the experiments, 100 CT scans were used from LIDC/IDRI dataset to test the proposed models.

#### 4.1.2. Extraction of Volume of Interest and Slice Selection

**Figure 10:**
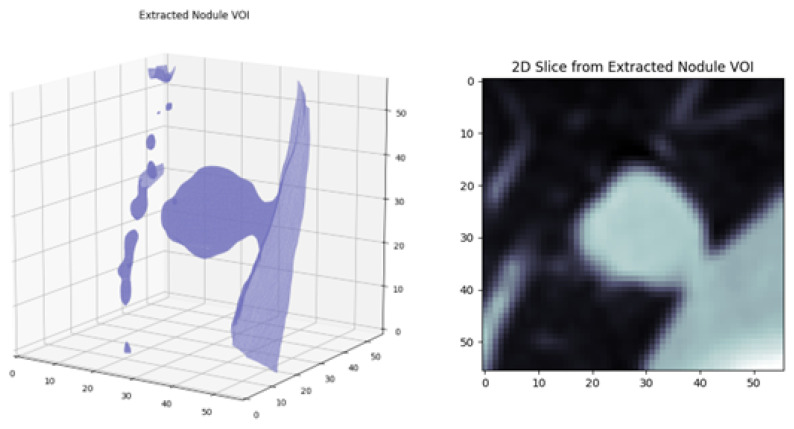
3D extracted volume of interest is given on the left and 2D transverse seen of the slice is given on the right.

In the annotation of nodules ≥ 3 mm, since each radiologist marks the surrounding boundary of the nodules, the volume center of the same nodule might differ from one radiologist to another. Therefore, as an initial step of extraction of VOI, the volume center of each annotated nodule is computed based on the provided annotations by each radiologist. If the center coordinates of nodules, annotated by different radiologists, are closer than the threshold, they are assumed to be the same nodule. Hence, at the next step, the average volume center for each nodule with the number of radiologists’ approval is found. A similar approach is used for detecting the average volume center and the number of radiologists’ approval for the non-nodules ≥ 3 mm. There is a possibility that some of the objects might be annotated as nodules by one radiologist and non-nodule by the other(s) or vice versa. To overcome this problem, once the average volume centers are computed for nodules and non-nodules, if the volume centers of nodules and the non-nodules are closer than the threshold, they are eliminated from the dataset. After the volume centers of the objects are determined, a 30 × 30 × 30 mm^3^ region around the volume center is extracted as the volume of interest. The reason for using a 30 × 30 × 30 mm^3^ bounding cube is that the longest axis of the annotated largest nodule can be 30 mm in the dataset as provided in [30]. In the LIDC-IDRI dataset, CT scans are collected from different CT scanners. Although all slices from all scans are 512 × 512 pixels, the physical size of a single pixel is not the same for all scans. Thus, a 30 × 30 × 30 mm^3^ bounding cube corresponds to different sizes of pixel resolution. However, the input data for training and testing the proposed MPF model should be the same size. Therefore, all extracted 30 × 30 × 30 mm^3^ are normalized to the maximum resolution of 56 × 56 × 56 pixels. Figure 10 shows a sample of 3D extracted volume of interest with its 2D transverse seen.

Since the bounding box is used and the nodules are not segmented out, there would be some slices which do not belong to the nodule within the extracted nodule volume of interest, and they should be removed from the nodule VOI. However, removing the slices which do not belong to the nodule may result in different sizes of the input data. For instance, one volume of interest can have 10 slices not belonging to the nodule and on the other hand, the other volume of interest can have 20 slices not belonging to the nodule, and removing these slices will cause different sizes of the input problem. To overcome this problem, the smallest nodule found within the data set and the number of slices belonging to that nodule are found. So, if we select the same number of slices to form each nodule VOI as the number of slices belonging to the nodule from the smallest nodule VOI, this guarantees that we will end up having the same number of slices in each nodule VOI, and all selected slices will belong to the nodule. In the dataset used in this dissertation, the smallest number of slices belong to the nodule found as 6. Hence, from each nodule VOI, 6 slices are selected from each perspective. These 6 slices can be selected in a different way. One way could be selecting 6 slices from the center of the VOI. However, in this approach, if the nodule size is big, then there is a high chance of ending up selecting similar slices, and this might be a disadvantage because they are not going to give any distinct information from slice to slice. Another approach that is used in this dissertation is selecting the slices from starting of the nodule to the end of the nodule by equal intervals. So that as much as distinct information from slice to slice is preserved.

### 4.2. Experimental Results of MPF Model

The dataset used to train and test the model is created using 100 CT scans from the LIDC/IDRI database, and it contains the nodules and non-nodules approved by at least one radiologist. Dataset is balanced, and there is a total of 604 nodule and non-nodule objects. Dataset is split into 2 parts, 70% for the training and 30% for the testing. Therefore, the training data has a total of 422 nodules and non-nodules, and the testing data has a total of 182 nodules and non-nodules.

**Figure 11:**
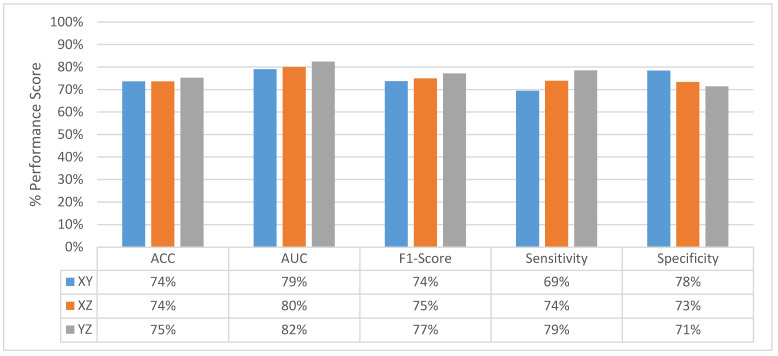
MPF model—change in slice-level classification performances across different perspectives.

**Figure 12:**
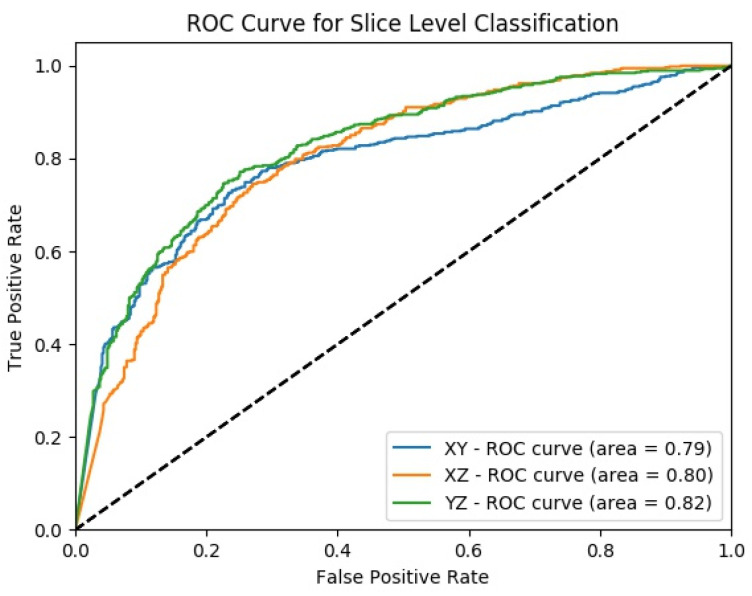
MPF model—ROC curves across different perspectives for slice-level classification.

**Figure 13:**
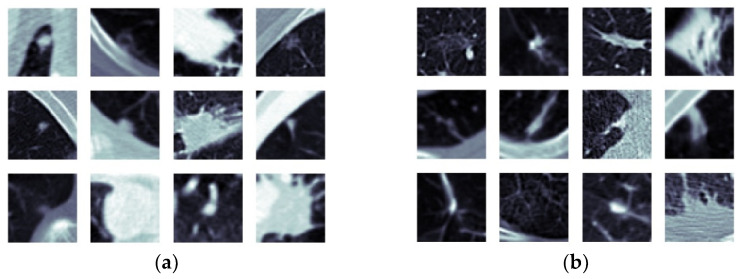
(**a**) MPF model—missed nodules (FN) and (**b**) MPF model—missed non-nodules (FP).

Figure 11 shows the change in slice-level classification performances across different perspectives for the MPF model which uses the raw slices from the extracted volume of interest. Although the slices from YZ-perspective give the highest ACC, AUC, F1-score, and sensitivity, the specificity of the model created using slices from YZ-perspective is the smallest. On the other hand, ACC, AUC, F1-score, and sensitivity of the model created using XY-slices are the smallest among all 3 models. However, the specificity of the model created using XY-slices is the highest among all 3 models. These results also can be interpreted as the model uses the slices from the YZ-perspective has a higher tendency towards type-I error and has higher FP. Alternatively, the model uses the slices from XY-perspective has a higher tendency towards type-II error and has higher FN. ROC curves across different perspectives for slice-level classification for the MPF model are shown in Figure 12. Missed nodules and non-nodules by the proposed MPF model are given in Figure 13.

**Figure 14:**
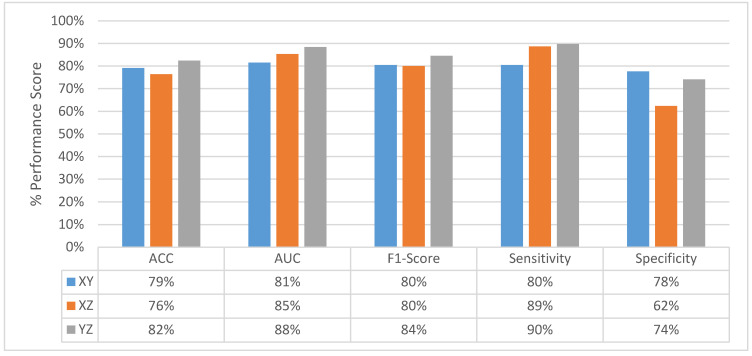
MPF model—change in perspective-level classification performances across different perspectives.

**Figure 15:**
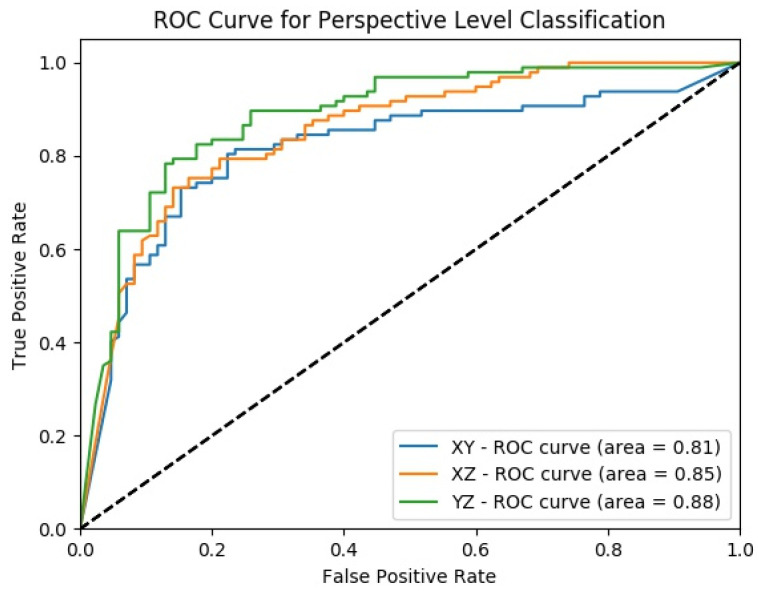
MPF model—ROC curves across different perspectives for perspective level classification.

After fusing the class scores from the slice level classification and obtaining the perspective level classification, except the specificity of the model uses the slices from XZ-perspective, all the other performance scores for all perspectives are increasing as shown in Figure 14. At the perspective level classification, while the model uses slices from XY-perspective still has the lowest type-I error and the highest type-II error, the tendency toward the type-I error of the model that uses YZ-perspectives is decreasing. At the perspective-level classification, still the model which uses the slices from the YZ-perspective has the highest performance score of ACC, AUC, F1-score, and sensitivity. ROC curves across different perspectives for perspective-level classification for the MPF model are shown in Figure 15.

**Figure 16:**
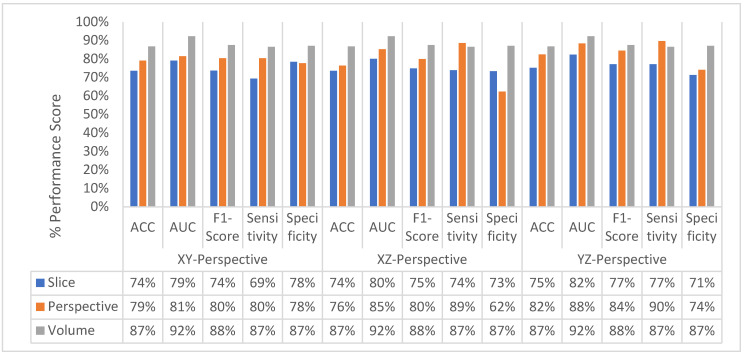
MPF model—Change in classification performance for slice, perspective, and volume-level classifications for each perspective.

The change in classification performance for slice, perspective, and volume level classifications for each perspective for the MPF model is shown in Figure 16. The increase in the classification performance from slice-level classification to perspective-level classification and volume-level classification can be seen in Figure 16. Slices-level classification gives the highest ACC at 75% using the slices from the YZ-perspective. When the class scores from multiple slices are fused at the perspective level, the highest classification ACC is increasing from 75% to 82%. Finally, adding another hierarchical fusion level which fuses the class scores from all perspectives increases the highest classification ACC from 82% to 87%. Similarly, AUC, F1-score, sensitivity, and specificity scores are also increasing from slice-level classification to perspective-level classification and volume-level classification. At the volume-level classification, both the tendency toward type-I error and type-II error are the same while having 87% sensitivity and specificity.

### 4.3. Experimental Results of SFMPF Models

In this study, four different SFMPF models based on Bilateral, Trilateral, Gabor, and LOG filters are experimented with using the same dataset which is used for the MPF model. In the SFMPF model, featured images are created by filtering the raw slices from the extracted volume of interest by the aforementioned filters. Once the featured image dataset is obtained for each proposed SFMPF model, the same approach as MPF is taken to create the model for slice-level classification, perspective-level classification, and volume-level classification.

#### 4.3.1. Experimental Results of SFMPF Model Based on Bilateral Image

**Figure 17:**
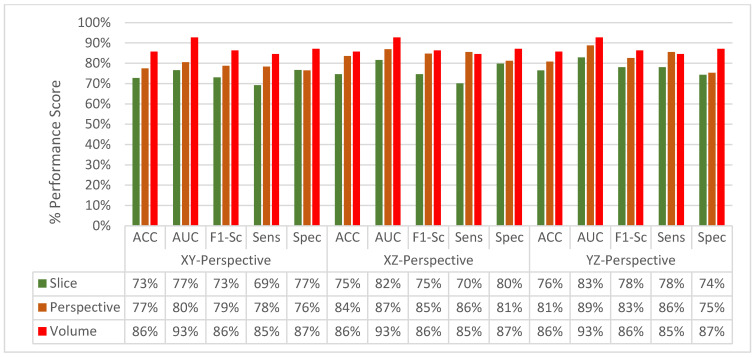
SFMPF model using Bilateral image—change in classification performance for slice, perspective, and volume-level classifications for each perspective.

**Figure 18:**
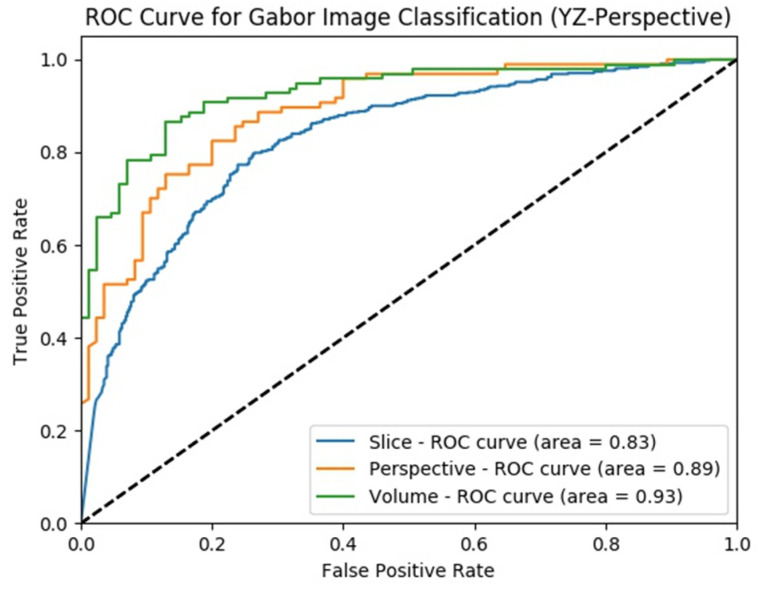
SFMPF model using Bilateral image—comparison of ROC curve for slice, perspective, and volume-level classifications for the slices from YZ-perspective.

**Figure 19:**
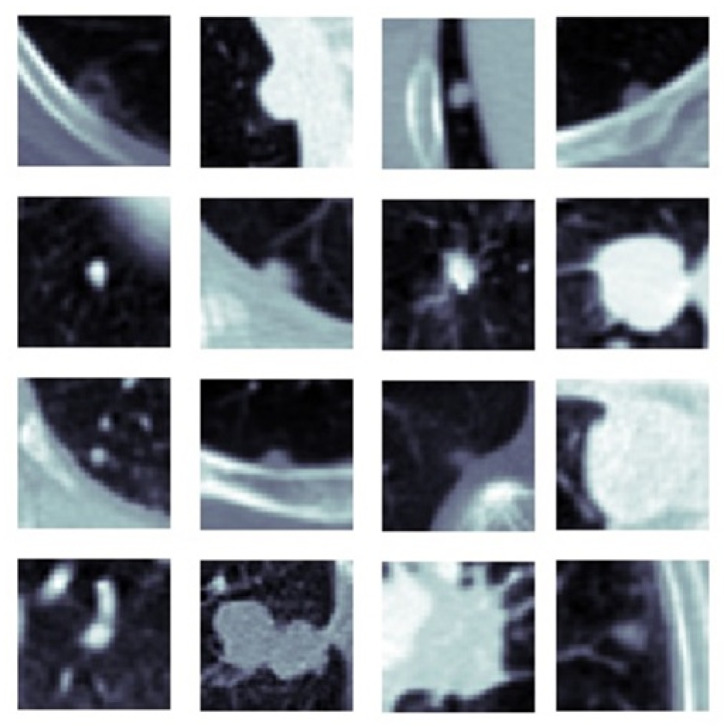
SFMPF model based on Bilateral image—missed nodules (FN).

**Figure 20:**
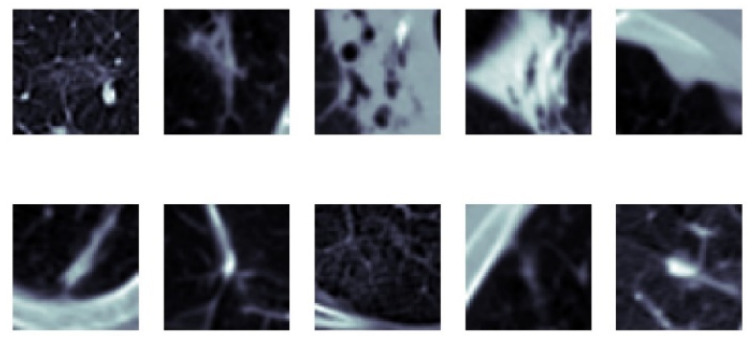
SFMPF model based on Bilateral image—missed non-nodules (FP).

Similar to the MPF model, an increase in the classification performance from slice-level classification to perspective-level classification and volume-level classification can be seen in Figure 17 for the Bilateral image-based SFMPF model. At the slice-level classification, the highest performance score of ACC, AUC, F1-score, and sensitivity achieved by the model uses the slices from the YZ-perspective. Compared to the MPF model, the Bilateral image-based SFMPF model accomplishes a slight improvement with respect to the highest ACC, AUC, F1-score, and specificity for the slice-level classification. However, at the perspective and the volume-level classifications, the MPF model achieves slightly better performance than the Bilateral image-based SFMPF for all of the performance measures except AUC. Since the highest AUC performance obtained from the model uses slices from YZ-perspective for the slice and the perspective-level classifications, a comparison of ROC curves for slice, perspective, and volume-level classifications for the slices from the YZ-perspective is provided in Figure 18. In addition, missed nodules and non-nodules by the proposed SFMPF model based on the Bilateral image are given in Figure 19 and Figure 20, respectively.

#### 4.3.2. Experimental Results of SFMPF Model Based on Trilateral Image

**Figure 21:**
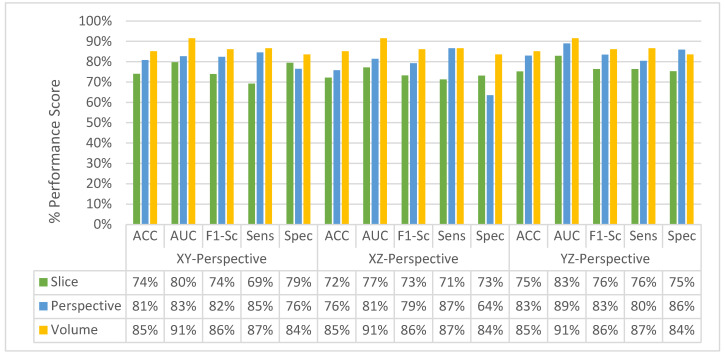
SFMPF model using Trilateral image—change in classification performance for slice, perspective, and volume-level classifications for each perspective.

**Figure 22:**
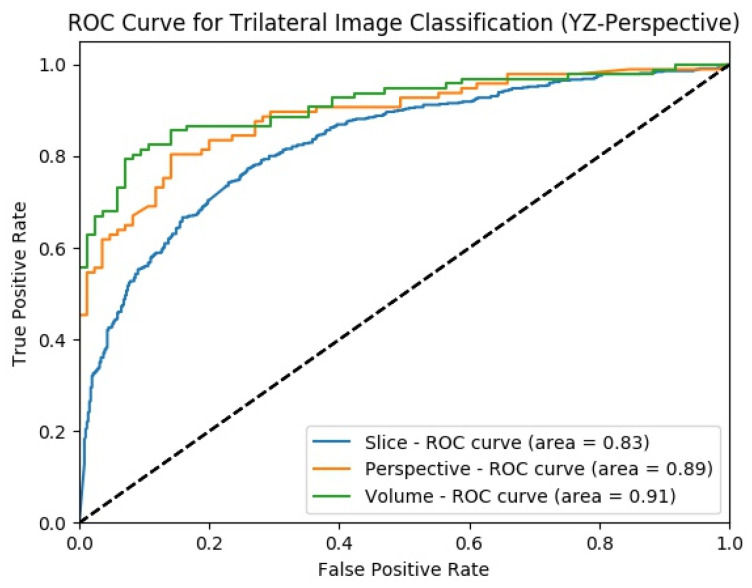
SFMPF model using Trilateral image—comparison of ROC curve for slice, perspective, and volume-level classifications for the slices from YZ-perspective.

**Figure 23:**
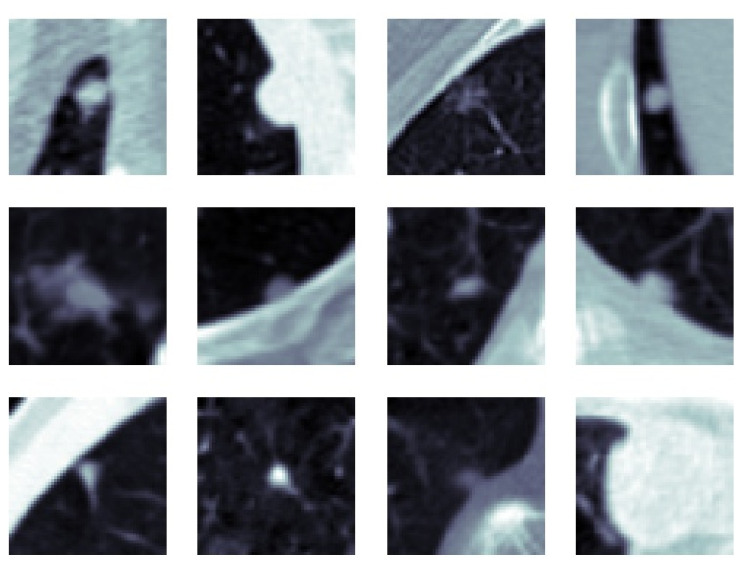
SFMPF model based on Trilateral image—missed nodules (FN).

**Figure 24:**
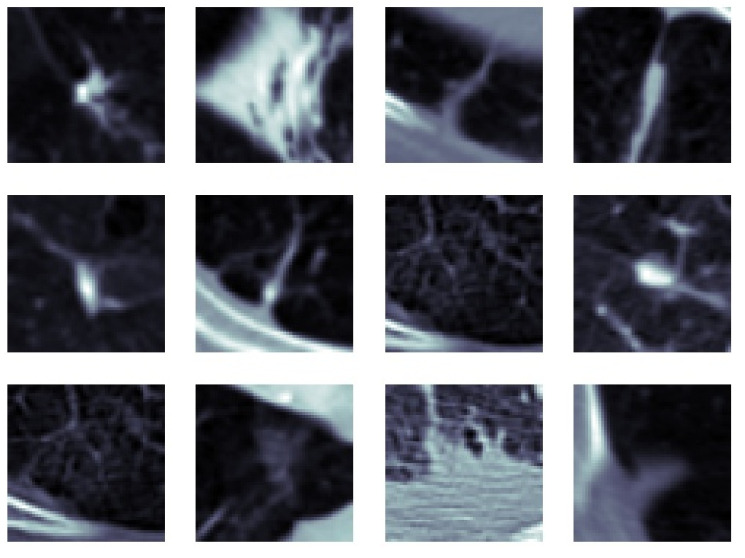
SFMPF model based on Trilateral image—missed non-nodules (FP).

The change in the classification performance for the slice, perspective, and volume-level classifications for each perspective is provided in Figure 21 for the Trilateral image-based SFMPF model. Throughout the hierarchical fusion, the highest performance scores are increasing from slice to volume-level classification such as ACC increases from 75% to 85%, AUC increases from 83% to 91%, F1-score increases from 76% to 86%, and sensitivity increases from 76% to 87%. The performance improvement from slice to volume-level classification also can be seen in Figure 22 which shows the comparison of the ROC curve for slice, perspective, and volume-level classifications. Moreover, the missed nodules and non-nodules by the proposed SFMPF model based on the Trilateral image are given in Figure 23 and Figure 24. Although the proposed hierarchical fusion approach works well with the Trilateral image-based SFMPF model, the overall performance of the MPF model achieves slightly better performance than the SFMPF model based on the Trilateral image. Tuning the parameters of the Trilateral filter such as spatial, range, and the Laplacian kernels’ standard deviation might improve the classification performance of the Trilateral image-based SFMPF model.

#### 4.3.3. Experimental Results of SFMPF Model Based on Gabor Image

**Figure 25:**
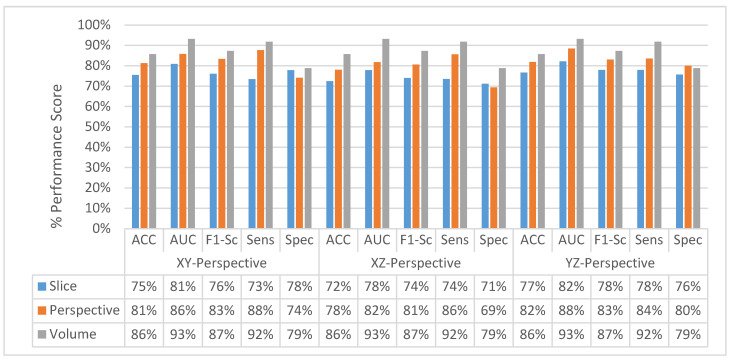
SFMPF model using Gabor image—change in classification performance for slice, perspective, and volume-level classifications for each perspective.

**Figure 26:**
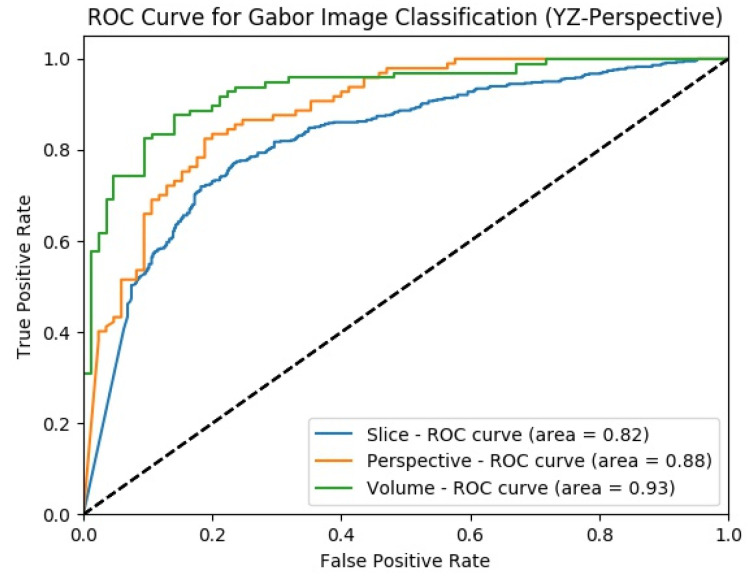
SFMPF model using Gabor image—comparison of ROC curve for slice, perspective, and volume-level classifications for the slices from YZ-perspective.

**Figure 27:**
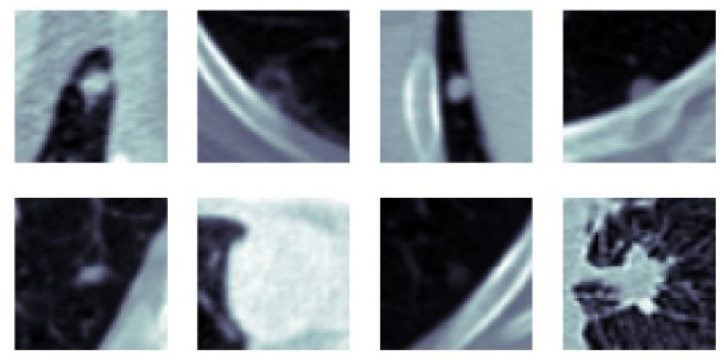
SFMPF model based on Gabor image—missed nodules (FN).

**Figure 28:**
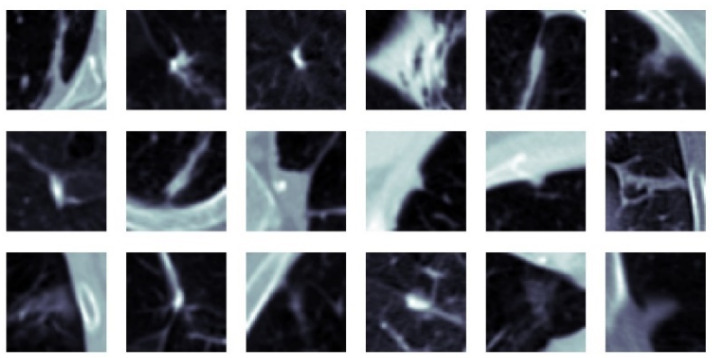
SFMPF model based on Gabor image—missed non-nodules (FP).

Classification performance improvement from the hierarchical fusion approach in the Gabor image-based SFMPF model can be seen in Figure 25 and the change in the ROC curve for slice, perspective, and volume-level classifications can be seen in Figure 26. In addition, the missed nodules and non-nodules by the proposed SFMPF model based on the Gabor image are given in Figure 27 and Figure 28. The highest ACC in the slice-level classification increases from 77% to 92% at the volume-level classification. While the highest sensitivity at the slice level increases from 78% to 92%, the highest specificity increases from 76% to 79% at the volume level. Hence, the Gabor image-based SFMPF model has a higher tendency toward type-I error compared to the MPF model. On the other hand, the Gabor image-based SFMPF model has higher sensitivity of 92% compared to the sensitivity of the MPF model which is 87%. Although, in the literature for texture extraction, the Gabor filter is used as a filter bank composed of multiple Gabor filters in different frequencies and angles, in the proposed Gabor image-based SFMPF model, a single Gabor filter is used to create the feature image. Using multiple Gabor filters with different frequencies and angles and then fusing them at the volume level may increase the performance of the proposed Gabor image-based SFMPF model.

#### 4.3.4. Experimental Results of SFMPF Model Based on LOG Image

**Figure 29:**
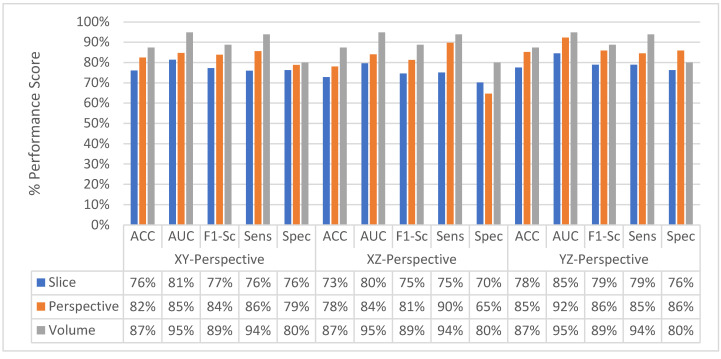
SFMPF model using LOG Image—change in classification performance for slice, perspective, and volume-level classifications for each perspective.

**Figure 30:**
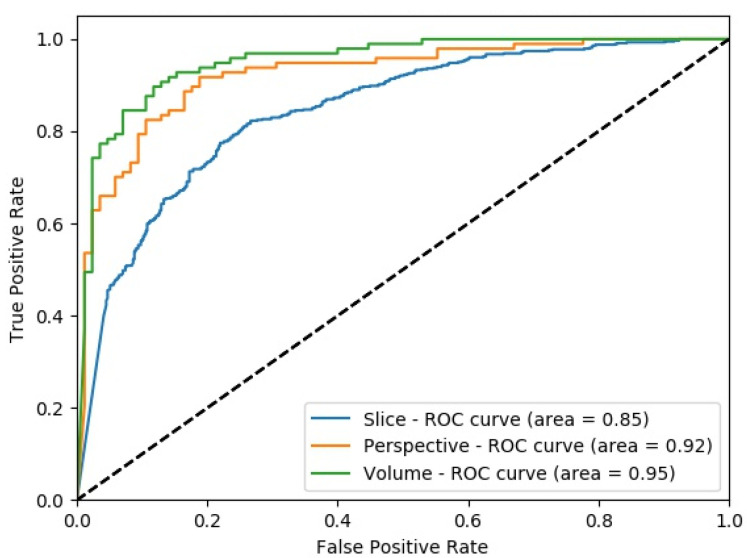
SFMPF model using LOG image—comparison of ROC curve for slice, perspective, and volume-level classifications for the slices from YZ-perspective.

**Figure 31:**
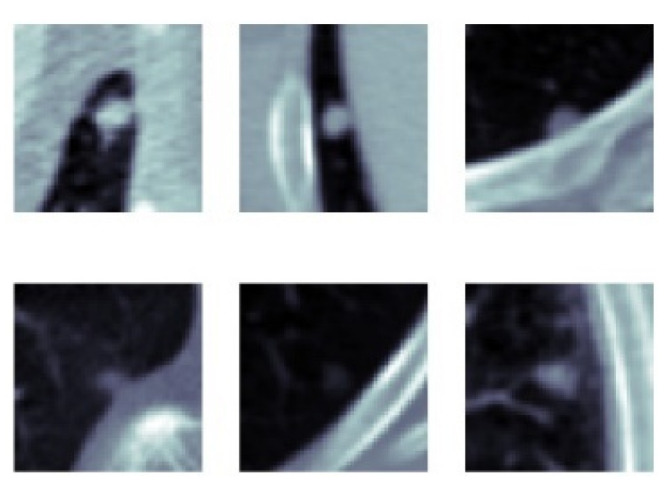
SFMPF model based on LOG image—missed nodules (FN).

**Figure 32:**
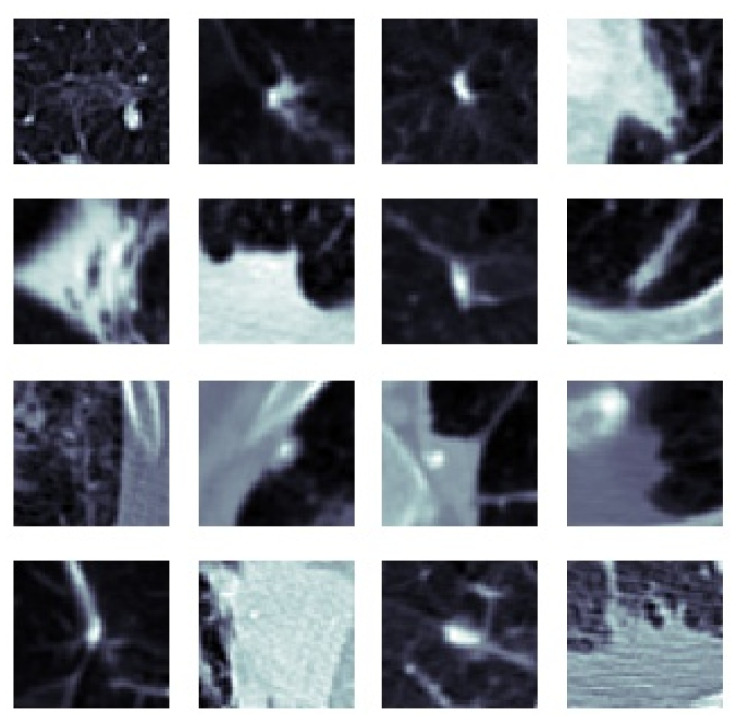
SFMPF model based on LOG image—missed non-nodules (FP).

Similar to other proposed SFMPF models and MPF models, the proposed hierarchical fusion-based deep learning approach significantly increases the performance of the classification result for the LOG image-based SFMPF model. The change in classification performance from slice to perspective and volume level classifications for each perspective is shown in Figure 29 and Figure 30. The highest ACC at slice-level classification increases from 78% to 85%, the highest AUC increases from 85% to 95%, and the highest sensitivity increases from 79% to 94% by hierarchically fusing the class scores from all perspectives at the volume level classification. LOG image-based SFMPF model achieves a sensitivity of 94% and a specificity of 80% at the volume level. Compared to the MPF model, it has higher sensitivity as well as a higher tendency toward type-I error. Missed nodules which are predicted as non-nodules (FN) by the LOG image-based SFMPF model are provided in Figure 31 and missed non-nodules which are classified as nodules (FP) are provided in Figure 32.

### 4.4. Classification Performance Comparison of SFMPF Models and MPF Model

In this section, the classification performances of the proposed feature image-based SFMPF models and the MPF model are compared with respect to ACC, AUC, F1-score, sensitivity, and specificity. First, each of the proposed SFMPF models is compared against each other, and then the performance of the MPF model is compared with the performance of the SFMPF models.

**Figure 33:**
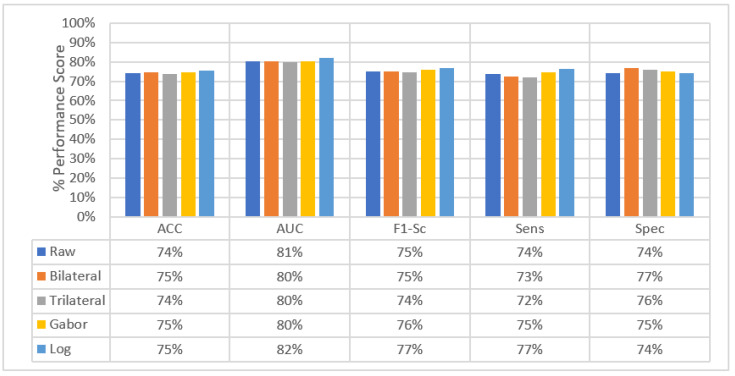
Change in average slice level classification performance of the proposed classifiers over three perspectives.

The change in the average slice-level classification performance of the proposed models over three perspectives is given in Figure 33. The Trilateral image-based SFMPF model has slightly lower performance compared to other feature image-based SFMPF models. On the other hand, the LOG image-based SFMPF model has the highest ACC, AUC, F1-score, and sensitivity among all. Whereas the Bilateral image-based SFMPF model has the highest specificity and lowest FPR. Both Bilateral and Trilateral image-based SFMPF models have a higher tendency toward type-II errors and higher false-negative rates (FNR) compared to Gabor and LOG image-based SFMPF models.

**Figure 34:**
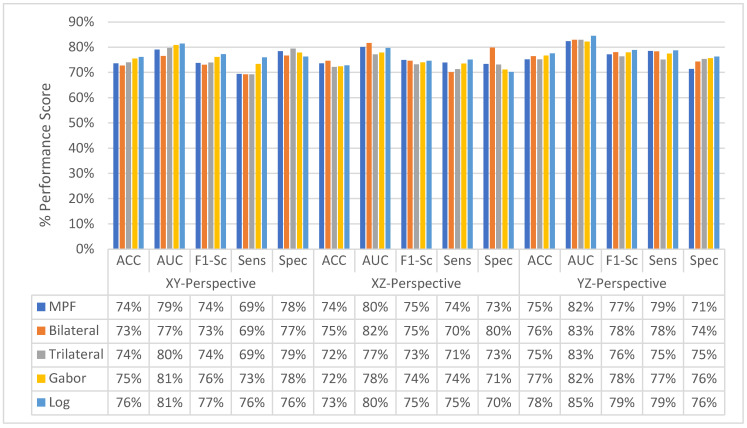
Change in slice-level classification performances for proposed SFMPF and MPF models for each perspective.

As shown in Figure 33, the proposed feature image-based SFMPF models improve the classification performance compared to the MPF model in terms of ACC, AUC, F1-score, sensitivity, and specificity. Particularly, the LOG image-based SFMPF model, while it increases the sensitivity compared to the MPF model, keeps the specificity the same. This shows that, whereas the LOG image-based SFMPF model increases the TPR, FPR remains the same. For a more detailed comparison, the change in the slice-level classification performances of the proposed models for each perspective is given in Figure 34.

**Figure 35:**
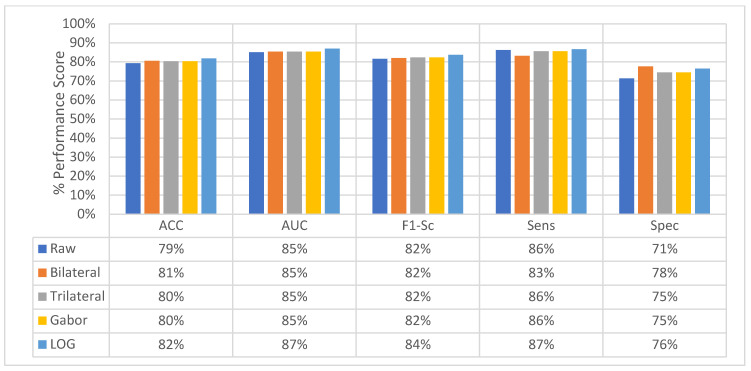
Change in average-perspective level classification performance of the proposed classifiers.

**Figure 36:**
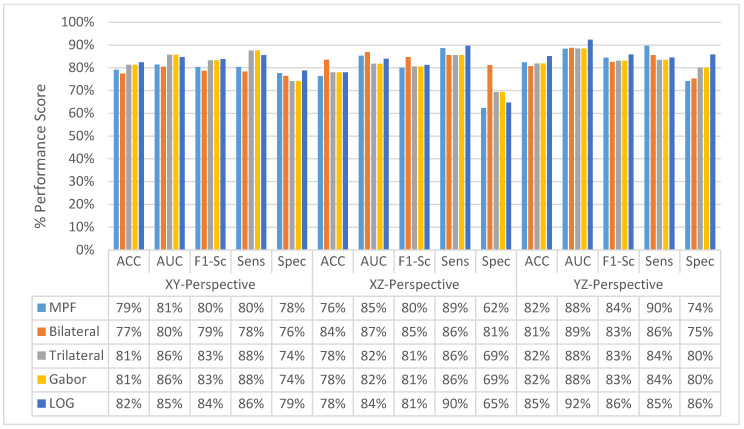
Change in perspective-level classification performances for each SFMPF model for each perspective.

At the second level, perspective level, in the proposed hierarchical fusion scheme, again the proposed feature image-based SFMPF models outperform the MPF model in terms of all performance measurements as depicted in Figure 35. At the slice-level classification, except the LOG image-based SFMPF model, all other proposed models either have the same sensitivity and specificity performances or higher specificity performances. In contrast to slice-level classification, all proposed models including the LOG image-based SFMPF model achieve higher sensitivity compared to specificity, and they all have a higher tendency toward type-II error at the perspective-level classification. This means that the nodule prediction performances of the proposed models are better than the non-nodule prediction performances at perspective-level classification. Particularly, the Bilateral image-based SFMPF model increases the sensitivity from 73% to 83% and specificity from 77% to 78% at the perspective level and LOG image-based SFMPF model increases the sensitivity from 77% to 87% and specificity from 74% to 76% at the perspective level. Whereas, the proposed MPF model improves the sensitivity from 74% to 86% and decreases the specificity from 74% to 71% at the perspective level. Therefore, one can conclude that the proposed feature image-based SFMPF models not only improve the sensitivity but also improve the specificity while the MPF model increases the sensitivity and decreases the specificity at the perspective level classification. For a more detailed comparison, the change in the perspective-level classification performances of the proposed models for each perspective is given in Figure 36.

**Figure 37:**
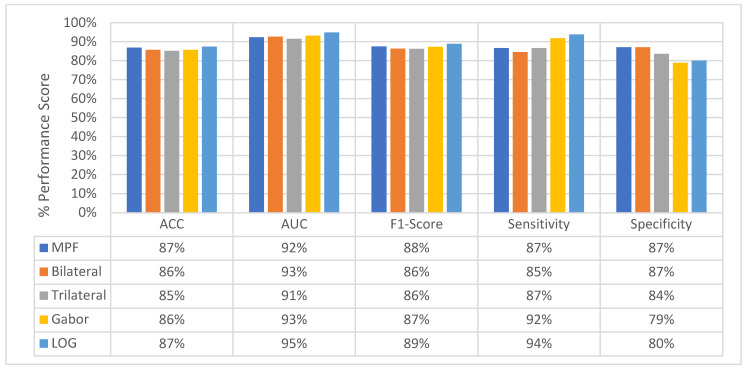
Change in volume-level classification performances for the proposed classifiers.

**Figure 38:**
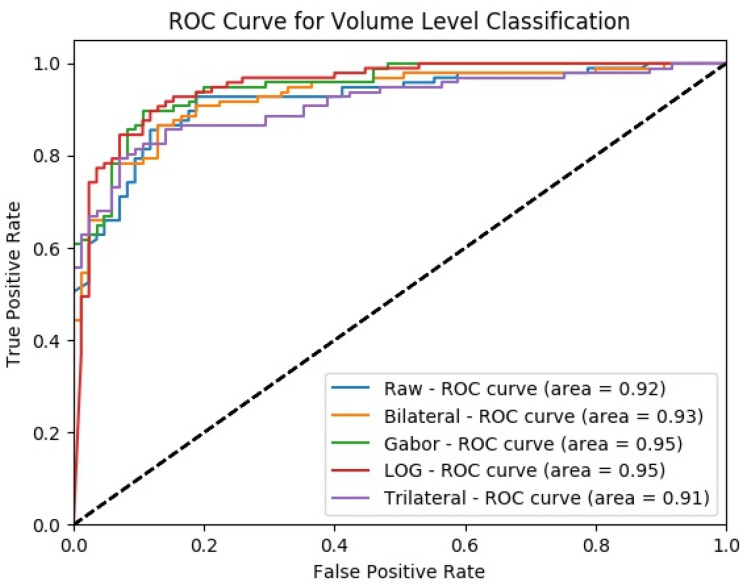
ROC curves from volume-level classification for each SFMPF model.

At the final level, volume level, classification, and the classification performances of all measures are increasing for all the proposed methods as seen in Figure 37 and Figure 38. The MPF model achieves a sensitivity and specificity of 87% at the volume level. If we compare the sensitivity and specificity performance of the first (slice) level and the last (volume) level classifications, the MPF model increases the sensitivity and specificity from 74% to 87%, the Bilateral image-based SFMPF model increases the sensitivity from 73% to 85% and specificity from 77% to 87%, Trilateral image-based SFMPF model increases the sensitivity from 72% to 87% and specificity from 76% to 84%, the Gabor image-based SFMPF model increases the sensitivity from 75% to 92% and specificity from 75% to 79%, and LOG image-based SFMFPF model increases the sensitivity from 77% to 94% and specificity from 74% to 80%. As seen from the results, at the final level of classification, while the LOG image-based SFMPF model predicts with the highest sensitivity, the MPF model predicts with the highest specificity. Similar to slice and perspective-level classification, the LOG image-based SFMPF model outperforms the other feature image-based SFMPF models as well as the MPF model with respect to AUC, F1-score, and sensitivity. Whereas the ACC, AUC, F1-score, and sensitivity increase through the proposed hierarchical fusion scheme, the tendency toward type-I and type-II errors of the proposed models varies from layer to layer.

### 4.5. Experimental Results of MFMPF Model

**Figure 39:**
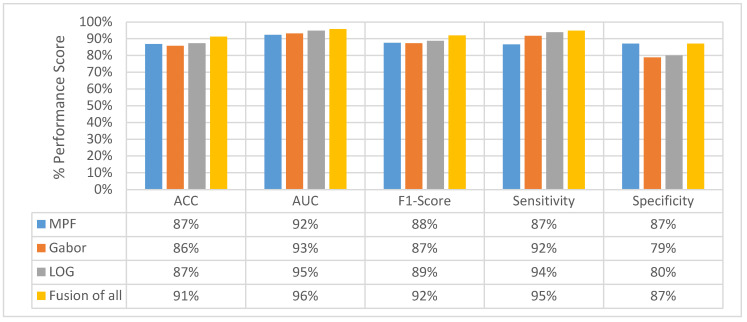
Change in final layer classification performance of MPF, SFMPF based on Gabor and LOG, and MFMPF models.

**Figure 40:**
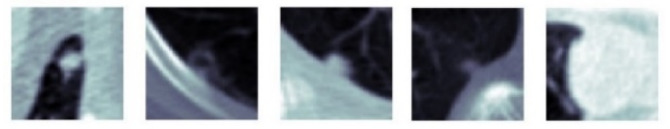
MFMPF model based on fusion of all—missed nodules (FN).

**Figure 41:**
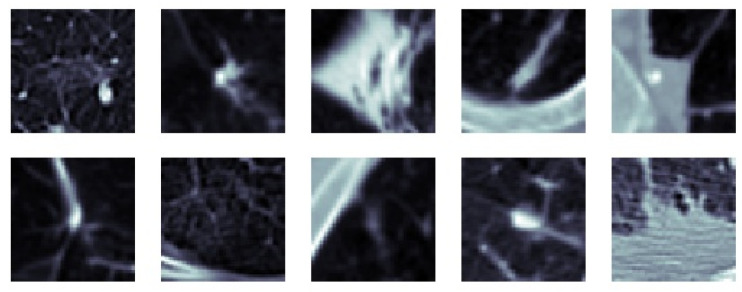
MFMPF model based on fusion of all—missed non-nodules (FP).

The idea behind the MFMPF model is the first to make multiple decisions for an object using different types of features by looking from different perspectives. Then, fusing each of the decisions made based on different features to make the final decision. Therefore, all previously proposed feature image-based SFMFP models and the basic MPF model which uses raw slices are fused to obtain the MFMPF model. By adding another hierarchy, the class scores obtained at the final (volume) layer of each SFMPF model are in the MFMPF model. Results from the MFMPF model with the MPF model and the best-performing SFMPF models based on Gabor and LOG images are given in Figure 39. The MFMPF model outperforms all proposed feature image-based SFMFP models as well as the MPF model with respect to all performance measures except specificity. Although the proposed MFMPF model does not perform better than the MPF model for detecting non-nodules, since it has higher sensitivity and the same specificity compared to MPF. We can conclude that while the TPR increases, FPR remains the same in the proposed MFMPF model and it performs better than all other proposed models. Missed nodules and non-nodules by the MFMPF model are provided in Figure 40 and Figure 41, respectively.

### 4.6. Performance Comparison of the Proposed Method with Relevant Studies

Table 1 provides the performance comparison of our prosed method with the state-of-the-art relevant studies with respect to the accuracy, sensitivity, specificity, and false positives per scan (fp/scan). The comparison table includes the studies that use the traditional nodule detection approaches using shallow classifiers as well as the state-of-the-art deep learning approaches. As seen in Table 1, the highest accuracy, sensitivity, and specificity scores were achieved by Choi et al. [10]. Choi et al. proposed a hierarchical 3D block-based lung nodules detection and classification method. In their proposed method, they used the 3D block analysis method to detect nodule candidates from CT scans. Then, after extracting the features from nodule candidates, they used SVM for classification and false positive reduction. The nodule candidate detection step in their proposed method introduces a very large number of false positives. They used 58 CT scans that contain a total of 151 nodules, and their proposed method detects a total of 3639 nodule candidates that include 147 nodules that are true positives and 3492 false positives. Then, they used SVM to reduce the false positives. Therefore, the nodule and non-nodules that are used to train the SVM classifier are defined by their candidate detection algorithm. However, our proposed method uses nodules and non-nodules which are annotated by radiologists as pulmonary lesions [30]. Although Choi et al. use the LIDC/IDRI dataset, the definition of negative samples (non-nodules) in their study and our study is different. Although Choi et al. reported the highest performance scores in terms of accuracy, sensitivity, and specificity, their proposed model has 2.27 fps/scan. Whereas our proposed method achieved a sensitivity of 95% with only 0.4 fps/scan. In addition, it is not clear how their proposed method achieved an accuracy of 97.61% while the sensitivity is 95.28% and specificity is 96.23%. Accuracy cannot be greater than both sensitivity and specificity at the same time since accuracy is a weighted average of them. In computer-aided detection algorithms, the aim is to increase the sensitivity of the model to detect all possible positive samples while decreasing the false positives. Therefore, according to the comparison table provided in Table 1, our proposed hierarchical deep-fusion learning scheme achieves very competitive and promising results compared to the state-of-the-art models in terms of the sensitivity and fp/scan.

## 5. Conclusions and Future Work

Lung cancer is the leading cancer type in terms of causing mortality in both men and women. As reported in previous studies, screening lung cancer using CT scans is a very common and effective method. However, detecting pulmonary nodules in CT scans is a very challenging problem, particularly for nodules in their early stages. CAD systems can be used by radiologists during the examination of CT scans to increase the nodule detection rate as well as to decrease false positives.

In this research, a hierarchical deep-fusion learning model is proposed for lung nodule detection from CT scans. Three different types of hierarchical deep-fusion learning models namely, multi-perspective deep-fusion learning (MPF) model, single-feature multi-perspective deep-fusion learning (SFMPF) model, and multi-feature multi-perspective deep-fusion learning (MFMPF) model, are proposed. The MPF model employs three levels of multi-perspective hierarchical deep-fusion-based classification. In the proposed model, each module at each level is trained separately in a hierarchical modular fashion; that is, the decision made at each level is predicted based on the decision from the previous layer. The final decision for each input of 3D volume of interest is made based on the predictions from multiple perspectives. To test the classification performance of the proposed MPF model, a total of 604 nodule and non-nodule objects are extracted from 100 CT scans, 70% of the data is used to train the proposed model and 30% of the data is used to test the proposed model. Experimental results show that the proposed hierarchical fusion-based deep learning model achieved an ACC of 74%, AUC of 81%, sensitivity of 74%, and specificity of 74% at the first, slice, level classification, ACC of 79%, AUC of 85%, sensitivity of 86%, and specificity of 71% at the second, perspective, level classification, and ACC of 87%, AUC of 92%, sensitivity of 87%, and specificity of 87% at the final, volume, level classification. As seen from the results, the proposed multi-perspective hierarchical fusion approach increases all the classification performance measures significantly from slice level to volume level.

In addition, a feature image-based hierarchical deep-fusion learning model called SFMPF is proposed. Four different feature image-based hierarchical deep-fusion learning models are explored by utilizing Bilateral, Trilateral, Gabor, and LOG-filtered images. Experimental results showed that using feature images instead of raw slices increases the classification performance at all levels. Particularly, the LOG image-based SFMPF model increases the AUC from 92% to 95%, and sensitivity from 87% to 94% compared to the MPF model at the volume-level classification. Finally, a multi-feature multi-perspective hierarchical deep-fusion learning model MFMPF is proposed by utilizing the predictions from the proposed four different SFMPF models. This additional level of hierarchical deep-fusion increases the ACC from 87% to 91%, AUC from 92% to 96%, F1-score from 88% to 92%, and sensitivity from 87% to 95% compared to the MPF model.

Although there is a significant classification performance improvement in the proposed multi-feature multi-perspective hierarchical deep-fusion learning approach, there are open problems and improvements waiting to be explored. One of the improvements that can be made to the model is 3D rotation invariance. If the nodule is not circular and elongated toward one of the axes, and if the rotated version of the similar nodule exists in another sample, most likely it is not classified as a nodule since DCNN is not rotation invariant. Therefore, making the proposed model robust to changes in the ratio can increase the classification performance. One way of making the proposed model invariant to rotation is normalizing the orientation of the input data to the same angle. This can be performed by fitting an ellipse to each input sample and finding the orientation of the elongated axis and normalizing them to the same angle. However, this proposed method requires a segmentation of the nodule and the non-nodule objects to be able to fit an ellipse to find the initial orientation of the object. In addition, different hierarchical fusion schemes can be explored utilizing feature image-based learning models. For instance, the fusion of feature image-based predictions at the slice or perspective level instead of at the volume level can be explored. In this study, the proposed MFMPF model synthesis the class scores at the volume level. However, different feature image-based predictions can be fused at the slice level based on their perspectives. Then, hierarchical deep-fusion learning can follow with the perspective and volume level fusions. Another improvement can be explored in the SFMPF model based on Gabor images. In the proposed SFMPF model based on Gabor images, only a single scale and orientation Gabor filter is used. To cover more structures with different orientations and scales, multiple Gabor filters can be used to create multiple Gabor image-based SFMPF models. Then, the final class scores from each model can be fused with an additional layer of the hierarchical classifier. Moreover, the long-short-term memory (LSTM) can be explored for classification after extracting the features with DCNN for slice-level predictions. LSTM is a type of recurrent neural network (RNN) which is used for prediction of the time-dependent sequence data such as video. For video content recognition, DCNN+LSTM-based deep learning has been explored in recent studies. In the CT scans, although there is no time dependency between the slices, there is a spatial correlation between the slices. Therefore, the DCNN+LSTM network can be explored for the proposed hierarchical deep-fusion learning scheme utilizing CT scans. Finally, even though the hierarchical deep-fusion learning scheme is proposed for lung nodule detection, the authors are planning to explore the proposed hierarchical approach for COVID-19 pneumonia detection using chest CT scans.

## Figures and Tables

**Table 1:** Comparison of the proposed hierarchical scheme with the relevant studies.

CAD System	Classification Method	Accuracy (%)	Sensitivity (%)	Specificity (%)	FPs/Scan
Our proposed method	Hierarchical Deep-Fusion	91.20	95	87	0.4
Haung et al., 2022 [31]	3 D CNN-TL	91.07	90.9	91.2	-
Jiang et al., 2021 [32]	3 D CNN-CBAM	90.77	85.3	95	-
Mastouri et al., 2021 [33]	Bilinear CNN	91.99	91.8	92.2	0.07
Zhai et al., 2020 [34]	MT-CNN	-	87.7	88.8	-
Liu et al., 2020 [35]	MMEL-3 D CNN	90.60	83.7	93.9	-
Ozdemir et al., 2020 [36]	3 D CNN	-	91	-	0.5
Pezeshk et al., 2019 [37]	3 D CNN	-	91	-	2
Monkam et al., 2018 [38]	Multi-patch CNNs	88.20	83.8	-	-
Rushil Anirudh et al., 2016 [19]	3 D CNN	-	80	-	10
A. A. Adiyoso Setio et al., 2016 [21]	Multi-view CNN	-	85.4	-	1
C. Jacobs et al., 2014 [15]	GentleBoost	-	80	-	1
W. J. Choi et al., 2013 [10]	SVM	97.61	95.28	96.23	2.27
D. Cascio et al., 2012 [9]	ANN	-	88	-	2.5
T. Messay et al., 2010 [8]	FLD	-	82.6	-	3
K. Murphy et al., 2009 [7]	k-NN	-	80	-	4.2

## Data Availability

The dataset that we used in our study is publicly available, and it can be found here: https://wiki.cancerimagingarchive.net/pages/viewpage.action?pageId=1966254 (accessed on 11 October 2022).
